# Influence of C_2_H_2_ Flow Rates on Optical Properties, Surface Roughness, and Residual Stress of Ti/WC Thin Films Deposited on Glass Substrates

**DOI:** 10.3390/ma18061269

**Published:** 2025-03-13

**Authors:** Chuen-Lin Tien, Yi-Lin Wang, Yuan-Ming Chang, Shih-Chin Lin, Ching-Chiun Wang

**Affiliations:** 1 Department of Electrical Engineering, Feng Chia University, Taichung 40724, Taiwan; m1305673@o365.fcu.edu.tw; 2 Ph.D. Program of Electrical and Communications Engineering, Feng Chia University, Taichung 40724, Taiwan; mason.jyishing@gmail.com; 3 Mechanical and Systems Research Laboratory, Industrial Technology Research Institute, Hsinchu 31057, Taiwan; shihchin@itri.org.tw (S.-C.L.); juin0306@itri.org.tw (C.-C.W.)

**Keywords:** Ti/WC thin film, pulsed DC magnetron sputtering, surface roughness, residual stress

## Abstract

This paper investigates the influence of C_2_H_2_ flow rates on the optical properties, surface roughness, and residual stress of Ti/WC thin films deposited on glass substrates. A range of Ti/WC thin films with varying carbon contents were prepared using the reactive pulsed DC magnetron sputtering technique. The properties of the Ti/WC films can be tuned by adjusting the deposition parameters, among which the acetylene (C_2_H_2_) flow rate plays a key role in determining the thin film’s microstructure, optical properties, and stress behavior. The optical properties of the thin films were analyzed using UV-visible-NIR spectroscopy and Fourier transform infrared (FTIR) spectroscopy, the surface morphology was analyzed using microscopic interferometry, and the residual stress in the films was measured using a homemade Twyman–Green interferometer. The measurement results show that the average reflectance of Ti/WC films decreases with the increase in the C_2_H_2_ flow rate, and the measured value changes from 52.24% to 44.56% in the wavelength of 400–800 nm. The infrared reflectance of Ti/WC films in the wavelength of 2.5–25 μm is 81.8% for 10 sccm, 80.8% for 20 sccm, 77.2% for 30 sccm, and 73.6% for 40 sccm. The tensile stress of the Ti/WC films deposited on B270 substrates increases with the increase in the C_2_H_2_ flow rate, and the stress value changes from 0.361 GPa to 0.405 GPa. The surface roughness of Ti/WC films initially increases and then decreases slightly with the increase in the C_2_H_2_ flow rate. These results indicate that the C_2_H_2_ flow ratio significantly affects the reflectance in the visible and infrared bands, surface roughness, and residual stress of the Ti/WC films, which is of great significance for optimizing thin film performance to meet specific application requirements.

## 1. Introduction

Amorphous diamond-like carbon (DLC) and tungsten carbide (WC) films are wear-resistant coatings with different properties, but they exhibit certain correlations in various applications, especially in terms of protective coatings, wear resistance, and mechanical properties. The amorphous diamond carbon (DLC) coating is renowned for its excellent properties, such as low friction coefficient, high hardness, and wear resistance [1]. The characteristic of DLC film is amorphous carbon film, which is composed of a mixture of amorphous carbon, including graphite (sp^2^ bonding) and generated diamond (sp^3^ bonding) structures [2]. The rich sp^3^ bonds contained in such dense DLC films can provide high hardness levels and low friction coefficients when used for tribology applications [3,4,5,6]. Common methods for coating DLC thin films include plasma-enhanced chemical vapor deposition (PECVD) [7], reactive magnetron sputtering deposition [8], high-power impulse magnetron sputtering (HiPIMS) [9], etc. Due to the extremely high density of carbon atoms per unit volume in DLC films, and under conditions of rapid growth and low substrate temperature, the bond angles of the carbon atoms that make up the film are severely distorted, resulting in significant residual stress in the thin film [10,11]. High residual stress is another challenge for DLC films, which leads to poor adhesion and even causes the film to peel off from the substrate during the deposition process. Thick DLC films produce intrinsic compressive stress that causes delamination of the film [12]. In addition, residual stress can be reduced by doping metallic/non-metallic elements, which effectively adjusts the microstructure, the adhesion, the proportion of sp^3^/sp^2^, and the surface roughness of DLC coatings [13]. Ouchabane et al. [14] studied the evolution of stress generated in DLC films of different thicknesses and their effect on the mechanical properties of the deposited films. However, there are not many studies on the optical properties and residual stress of WC thin films by reactive magnetron sputtering deposition. Another significant feature of WC is its extremely high elastic modulus, which can only be exceeded by that of diamond [15].

Tungsten carbide (WC) has various specific properties, including extreme hardness, low friction coefficient, oxidation resistance, chemical inertness, and excellent thermal stability [16,17]. WC thin films can be prepared by various coating processes, such as plasma spraying [18], chemical vapor deposition (CVD) [19,20], and various magnetron sputtering techniques [21,22]. Tungsten carbide has various applications due to its good mechanical properties, such as fracture toughness, fatigue, and creep resistance. WC thin films are used as wear-resistant coatings, while titanium/tungsten carbide (Ti/WC) thin films are recognized for their excellent mechanical and optical properties, making them valuable for applications in optoelectronics, protective coatings, and wear-resistant materials. Reactive pulsed DC magnetron sputtering (PDMS) has become an effective deposition technique for preparing Ti/WC thin films due to advantages such as fast deposition rate, good film uniformity, and easy control of film composition by adjusting process parameters. One of the key parameters influencing the growth of Ti/WC films is the flow rate of acetylene (C_2_H_2_), which serves as the carbon precursor. Adjusting the flow rate of C_2_H_2_ will affect the carbon content in the Ti/WC thin films and thus change the microstructure of the thin film, resulting in changes in its surface morphology and optical and mechanical properties. The connection between the DLC and Ti/WC coatings is due to the deposition of Ti/WC thin films through reactive pulsed DC magnetron sputtering, which is also commonly used for DLC thin film deposition. In addition, both DLC and Ti/WC coatings are affected by similar deposition parameters, such as C_2_H_2_ flow rate, which can affect the carbon content and final performance of the thin films. Through comparison with DLC thin film, this study explores how the addition of carbon to Ti/WC films through the flow of C_2_H_2_ affects properties such as hardness, optical properties, and residual stress behavior, just as it does in DLC coatings. However, the properties of these DLC coatings may be adversely affected by residual stresses generated during the deposition process. Therefore, understanding the role of film residual stress in wear resistance is crucial to optimizing the industrial applications of these coatings.

Thin film coatings exhibit internal residual stress owing to the growth process of the thin film deposited on the substrates. Residual stress in thin films arises from various factors, including differences in thermal expansion coefficients, phase transformations, and deposition techniques. These residual stresses can lead to microstructural changes, affecting the mechanical properties and overall performance of the thin films. Previous studies have shown that optical interferometry can be used to accurately measure residual stress in thin films and determine the nature (tensile or compressive) and magnitude of residual stress [23,24]. At present, there is little research on the residual stress and thermal stress of Ti/WC thin films deposited on glass substrates. This study aims to focus on the optical and mechanical properties, residual stress, and surface roughness of Ti/WC thin films deposited on B270 and H-K9L glass substrates. Both B270 and H-K9L substrates are optical glasses with similar refractive indices, but H-K9L glass is typically preferred for its higher optical performance due to slightly better optical clarity and transmission properties. B270 glass is more commonly used in optical applications where cost-effectiveness is also important. It can also help optimize deposition parameters to enhance the performance of Ti/WC coatings.

## 2. Materials and Methods

### 2.1. Preparation of Ti/WC Thin Films

**Figure 1:**
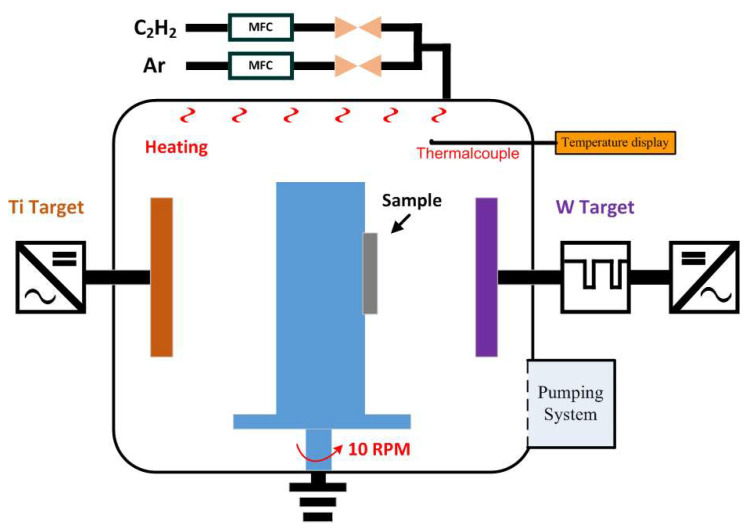
Schematic diagram of reactive pulsed DC magnetron sputtering system.

This study used a reactive pulsed DC magnetron sputtering (PDMS) system (Jyi Shing Technology Co., Ltd., Taichung, Taiwan) to prepare Ti/WC (titanium/tungsten carbide) thin films, as shown in Figure 1. The reactive PDMS not only suppresses the arcing effectively, but it may also have potential advantages in terms of controlling the reactive sputtering and film composition without tuning the reactive gas [25]. In the pulsed DC magnetron sputtering process, by the collision of positive ions, a positive charge accumulates on the surface of the target material. This results in a decrease in the tendency of positive ions toward the target material. Our PDMS system was equipped with two 250 × 50 mm^2^ rectangular target materials. A flat tungsten target and titanium target were sputtered in the Ar/C_2_H_2_ gas mixture condition. A thermocouple mounted close to the coating substrate position was used to detect the deposition temperature. A thermocouple is a common device used to measure temperature by exploiting the thermoelectric effect, where two different metal wires are joined at one end and exposed to a temperature gradient. When the wire connection experiences a temperature difference, a small voltage (Seebeck voltage) is generated, which can measure the temperature inside the vacuum chamber. Two kinds of well-polished glass substrates one inch in diameter were cleaned in a mixture of acetone and isopropanol using an ultrasonic bath and blow-dried with N_2_ gas prior to being inserted into the deposition chamber. The preparation process of pulsed DC magnetron sputtering (PDMS) has the characteristics of low deposition temperature and high deposition rate. However, tungsten carbide (WC) film deposited directly on a glass substrate using the PDMS technique presents adhesion issues [26], resulting in low film hardness and easy detachment from the substrate. This may be because the coefficient of thermal expansion (CTE) of tungsten carbide is different from that of glass. When the deposited film undergoes temperature changes (such as during the cooling process after deposition), the expansion or contraction of the material will generate stress at the interface. This problem can be solved by applying a coating of Ti metal film as an intermediate layer between the glass substrate and the WC thin film. In general, the WC thin film coatings are used in industries as protective coatings due to high hardness and wear resistance. The PDMS system used a DC power supply and a pulsed DC power supply to provide power to the targets. The sputtering test sample was placed on a rotating base between the targets. The rotating base rotated at a speed of 10 rpm, which can control the revolution and rotation to deposit thin films of different thicknesses. Firstly, a rectangular titanium target material was used, and argon gas was introduced, which was powered by a DC power supply for the deposition of titanium thin films. A tungsten (W) target material was then used, and argon gas introduced through a medium-frequency (MF; T_on_ 25 μs/T_off_ 25 μs) power supply. Before each deposition, the chamber was evacuated to approximately 1.5 × 10^−5^ Pa, by a combination of rotary and turbo-molecular pump systems. The sputtering was carried out by using high-purity argon as sputtering gas. The mass flow controller (MFC) was used to adjust the flow rate of acetylene gas (C_2_H_2_) to sputter tungsten carbide (WC) films with different target poisoning ratios onto a glass substrate. The coating parameters, including substrate temperature, power density, process pressure, deposition time, and Ar flow rate were fixed except for the gas flow rate of C_2_H_2_ (10, 20, 30, and 40 sccm). Table 1 lists the sputtering conditions for the deposition of Ti/WC thin films in this study. The coating conditions of the four Ti/WC thin film samples are as follows: The metal Ti thin film coating conditions are an Ar flow rate of 60 sccm, coating time of 60 min, using a DC power supply with a power of 1.9 kW and a power density of 3.2 W/cm^2^. The bilayer process conditions for Ti/WC thin film are an Ar flow rate of 60 sccm and a C_2_H_2_ flow rate of 10 sccm. The first Ti/WC (titanium/tungsten carbide) sample is numbered A6C1, the processing time is Ti 40 min/MF 40 min, the working pressure is 0.25 Pa, the power density is 3.2 W/cm^2^ for Ti film and 6.4 W/cm^2^ for WC thin film. The second coating conditions for WC thin film are an Ar flow rate of 60 sccm and a C_2_H_2_ flow rate of 20 sccm. The sample number is A6C2, the processing time is Ti 40 min/MF 40 min, the working pressure is 0.25 Pa, the power density is 3.2 W/cm^2^ for Ti film and 6.4 W/cm^2^ for WC thin film. The third coating conditions for WC thin film are an Ar flow rate of 60 sccm and a C_2_H_2_ flow rate of 30 sccm. The third Ti/WC sample is A6C3, the processing time is Ti 40 min/MF 40 min, the working pressure is 0.25 Pa, the power density is 3.2 W/cm^2^ for Ti film and 6.4 W/cm^2^ for WC thin film. The fourth coating conditions for WC thin film are an Ar flow rate of 60 sccm and a C_2_H_2_ flow rate of 40 sccm. The fourth Ti/WC sample is A6C4, the processing time is Ti 40 min/MF 40 min, the working pressure is 0.25 Pa, the power density is 3.2 W/cm^2^ for Ti film and 6.4 W/cm^2^ for WC thin film. Furthermore, thin film coating experiment controls the deposition conditions of Ti and WC thin films through different C_2_H_2_ flow rates to obtain different Ti/WC film properties and further improves the sputtering effect of thin films using the thin film stress measuring technique. Ti/WC thin films were simultaneously deposited on B270 and H-K9L glass substrates using the PDMS technique. The deposition conditions, including power, pressure, and temperature were kept constant, and only the C_2_H_2_ flow rate was varied to create thin films with different residual stress states.

### 2.2. Residual Stress Measurement

**Figure 2:**
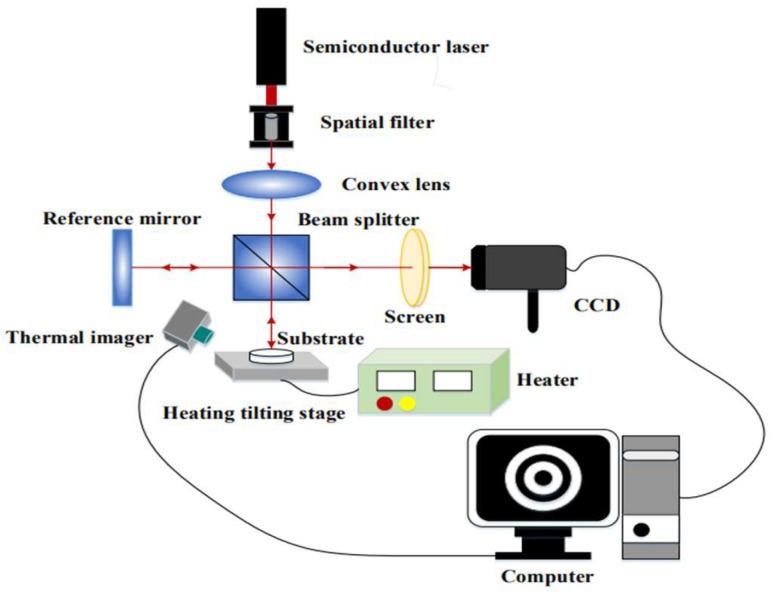
Schematic diagram of the measurement system for measuring residual stress and thermal stress.

We used a homemade Twyman–Green interferometer to measure residual stress in thin films [23,24]. The improved interferometer was equipped with a heating stage and an NIR thermal imager, as shown in Figure 2. A helium–neon laser with a wavelength of 633 nm was used as the light source. The laser beam passes through a spatial filter to form a point source. Then the light passes through a collimating lens to generate a uniform parallel beam and is split into two beams by a beam splitter. After being reflected by the reference mirror and the surface of the test sample, the two light beams are recombined into one light beam by a beam splitter, forming interference fringes on the rotating screen. The interference pattern is photographed by a CCD camera (acA 1300, BASLER, Ahrensburg, Germany), and the surface profile of the thin film is reconstructed through fast Fourier transform and phase recovery methods to determine the curvature radius of the test object. Finally, we calculate the residual stress in the thin film using Stoney’s formula [27].

The film thickness t_f_ of the coatings was measured by an alpha-step stylus profiler (D-500, KLA-Tencor, Chandler, AZ, USA). The residual stress was calculated by the change in the radius of curvature of the glass substrate before and after deposition. The Stoney formula was used to estimate the residual stress, which is given as:

(1) $$\sigma =\sigma_{i}+\sigma_{th}=\frac{1}{6}\frac{E_{s}}{\left(1-v_{s}\right)}\frac{{t_{s}}^{2}}{t_{f}}\frac{1}{R},\frac{1}{R}=\frac{1}{R_{2}}-\frac{1}{R_{1}},$$

where *σ* is the residual stress in the thin films; *σ_i_* is the internal stress; *σ_th_* is the thermal stress; *E_s_* represents Young’s modulus of the substrate; *ν_s_* is the Poisson’s ratio of the substrate material; *t_s_* is the thickness of the substrate; *t_f_* is the thickness of the film; and *R*_1_ and *R*_2_ are the curvature radius values of the substrate before and after the coating process, respectively. *R* represents the change in the curvature radius of the substrate before and after thin film coatings. The thermal stress (*σ_th_*) originates from the mismatch in thermal expansion between the thin film and the substrate. The thermal stress can be expressed as follows:

(2) $$\sigma_{th}=\left(\alpha_{s}-\alpha_{f}\right)\frac{E_{f}}{1-\nu_{f}}\left(T_{2}-T_{1}\right),$$

where α_f_ and α_s_ represent the thermal expansion coefficients of the thin film and substrate, respectively, and E_f_ and *ν_f_* are the Young’s modulus and Poisson’s ratio of the thin film.$\frac{E_{f}}{1-v_{f}}$ is the biaxial modulus of the thin film. *T*_1_ and *T*_2_ are the temperatures of thin films before and after heating, respectively. Table 2 lists the physical parameters of different glass substrates.

## 3. Results and Discussion

### 3.1. Optical Properties of Ti/WC Thin Film in Visible Light and Infrared Spectra

**Figure 3:**
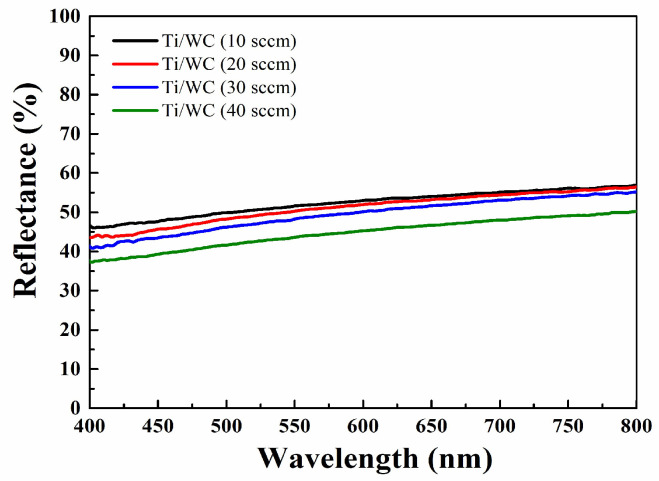
The average reflectance of Ti/WC thin films at different C_2_H_2_ flow rates.

This research employed a UV-VIS-NIR spectrophotometer (UV-2600i, Shimadzu Corporation, Kyoto, Japan) to measure the spectra in the wavelength range of 200 nm to 1000 nm. The optical properties of a sputtered Ti/WC (titanium/tungsten carbide) thin film are influenced by both the materials involved and their interactions across different wavelengths in the visible and infrared (IR) spectra. In the visible range (400–800 nm), titanium (Ti) is known to be reflective in the visible range. Tungsten carbide is generally a refractory material with high density and high melting point, which tends to be highly reflective in the visible light range. The four Ti/WC thin film thicknesses are 1.02 μm, 1.37 μm, 1.58 μm, and 1.78 μm. The average reflectance values in the wavelength range of 400–800 nm are 52.24% for 10 sccm, 51.01% for 20 sccm, 49.26% for 30 sccm, and 44.56% for 40 sccm. The average reflectance decreases with the increase in the C_2_H_2_ flow rate, and its value changes from 52.24% to 44.56%, as shown in Figure 3. In the visible range (400–800 nm), Ti/WC thin films generally show a high reflectance and low transmittance due to the metallic nature of titanium and the high reflectance of tungsten carbide.

Both titanium and tungsten carbide have complex refractive indices in the visible and IR ranges, meaning they have both real and imaginary (describing the absorption of light) components. The refractive index of titanium typically increases in the visible range (around *n* = 2.4–2.7). This can vary depending on the specific surface oxidation. We used a spectroscopic ellipsometer (M-2000, J. A. Woollam, Lincoln, NE, USA) to study the optical constants of thin film materials. It was found that as the C_2_H_2_ flow rate increases, the refractive indices (n) of Ti/WC films are 2.749 for 10 sccm, 2.614 for 20 sccm, 2.525 for 30 sccm, and 2.566 for 40 sccm. The measurement results show that the refractive index of Ti/WC thin film decreases as the C_2_H_2_ flow rate increases from 10 sccm to 30 sccm but increases slightly at 40 sccm. The average refractive index of the four Ti/WC films is *n* = 2.614 in the visible wavelength range of 400–800 nm. However, this can also vary with its crystallographic form and film thickness.

**Figure 4:**
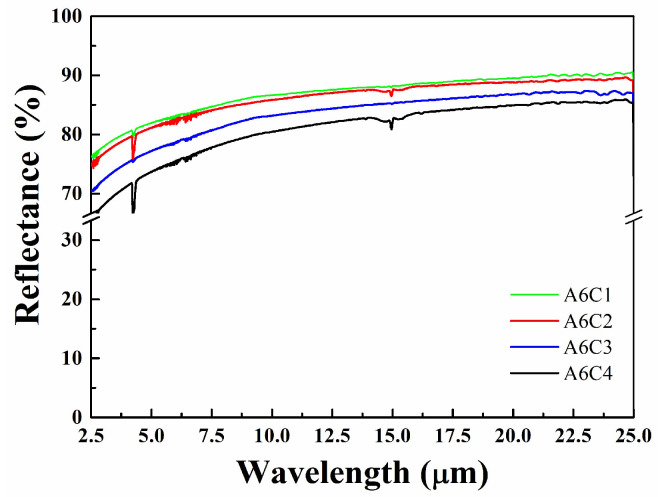
FTIR reflectance spectra of Ti/WC thin films at different C_2_H_2_ flow rates.

The infrared reflectance of Ti/WC thin films was evaluated using a Fourier transform infrared spectrometer (Thermo Fisher Scientific, Waltham, MA, USA). In the infrared (IR) wavelength range, titanium is generally reflective, especially at longer wavelengths. Its reflectance increases as the wavelength shifts into the IR region, due to its metallic nature. Tungsten carbide is also highly reflective in the IR range, and depending on its crystallinity, it may show a varied reflectance level at different IR wavelengths. Since Ti/WC bi-layer thin films are composed of metal and dielectric thin films, the thickness is relatively thick, and the transmittance in the visible light and infrared bands is relatively low. Moreover, they may scatter light, especially in cases where the film surface is rough or porous. The combination of Ti and WC can produce thin films that have specific characteristics for light manipulation, such as selective absorption depending on the deposition conditions. Both materials (Ti and WC) are typically opaque in the visible and infrared ranges due to their metallic or ceramic nature, which means they do not exhibit significant transparency. The optical properties of sputtered films are strongly dependent on their thickness. Figure 4 demonstrates that the infrared reflectance of Ti/WC films decreases with the increase in the C_2_H_2_ flow rate. The infrared average reflectance of Ti/WC films in the wavelength of 2.5–25 μm are 81.8% for sample A6C1 (C_2_H_2_ flow rate of 10 sccm), 80.8% for sample A6C2 (C_2_H_2_ flow rate of 20 sccm), 77.2% for sample A6C3 (C_2_H_2_ flow rate of 30 sccm), and 73.6% for sample A6C4 (C_2_H_2_ flow rate of 40 sccm). Thin films may exhibit interference effects, while thicker films behave more like bulk materials, with higher reflectance and less scattering. In the infrared range, both titanium and tungsten carbide are highly reflective, with the potential for selective absorption and scattering depending on the microstructure of the sputtered film. The specific optical properties of the sputtered Ti/WC thin films in the visible and IR spectra can be fine-tuned by adjusting the deposition parameters (e.g., flow rate, sputtering power, pressure, and substrate temperature) to control the phase composition, microstructure, residual stress, and surface morphology of the thin films. Absorption is related to thin film thickness. The Ti/WC thin films also demonstrate a low absorption rate of ~5% in the infrared band (2.5–25 μm).

### 3.2. Raman Spectra of Ti/WC Thin Films

Raman spectroscopy is a non-destructive chemical analysis technique that provides detailed information about chemical structure, phase and polymorphism, crystallinity, and molecular interactions. It is based upon the interaction of light with the chemical bonds within a material. Raman spectra consist of multiple peaks that show the intensity and wavenumber position of Raman scattered light. Each peak corresponds to a specific molecular bond vibration, including individual bonds and groups of bonds. Therefore, it is a powerful tool for studying the structural and chemical properties of materials, including thin films such as Ti/WC sputtering films. The most commonly used method is excitation using laser wavelengths in the ultraviolet and visible light regions. When studying Ti/WC films by Raman spectroscopy, the technique provides information on crystallinity, phase composition, and any defects or stresses that may be present in the material. The Raman spectrum can distinguish between different titanium (Ti) phases and tungsten carbide (WC). For instance, titanium could exhibit peaks related to the characteristic Ti–Ti modes. The difference in measurement lies in the wavelength of the light source. This study utilized near-infrared (785 nm, 1.58 eV) excitation that is particularly sensitive to sp^2^ carbon. The WC film typically shows distinct Raman peaks, such as the strong peak around 1100 cm^−1^, which is associated with the C-W-C bending or stretching modes. It is typically attributed to the C–C stretching vibration within the carbide structure. The peak can vary slightly depending on the quality and stoichiometry of the WC film, since the structure of Ti/WC thin films is a mixture of sp^2^ and sp^3^ hybridization sites. The low frequency carbon–carbon (C–C) vibrations occur around 800 cm^−1^. The vibrations of two carbon atoms are linked by strong double bonds (C=C) above 1600 cm^−1^. This strong peak is at a higher frequency than two carbon atoms connected by a weaker single bond (C–C, at 800 cm^−1^).

**Figure 5:**
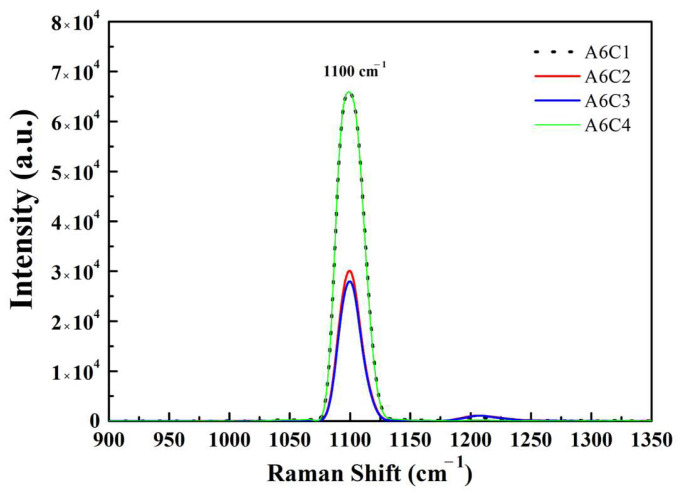
Raman spectra of Ti/WC thin films.

Figure 5 shows the Raman spectra of Ti/WC thin films. The intensity of the microcrystalline diamond mode is indicated by a Raman peak at 1100 cm^−1^, which is close to the 1140 cm^−1^ value reported in [28]. The frequency of the graphite mode changes to low frequency, likely due to an increase in internal tensile stress. The Raman study reveals that the peak at 1150 cm^−1^ is due to sp^3^ bonded carbon appearing with a CO additive [29,30]. The spectra corresponding to Figure 5 do not exhibit the diamond peaks centered at 1323–1330 cm^−1^, together with peak features at around 1500 cm^−1^ corresponding to the G-band wavenumber. The strong Raman peak shifts to a lower wavenumber compared with the 1332 cm^−1^ observed for natural diamonds and has a symmetric line shape toward the lower wavenumbers due to the small crystallite size or nanocrystallite/amorphous structure. Since DLC coatings are known for their extremely high hardness and low friction properties, they are an ideal choice for reducing wear and extending the service lifetime of coated parts. This study mainly focuses on the preparation of Ti/WC coatings. Ti/WC thin film is designed to enhance its hardness and wear resistance and achieve certain characteristics to replace the DLC layer. This situation is due to the presence of carbon in the Ti/WC films and specific conditions for depositing the required thin film, such as controlling the C_2_H_2_ flow rates during the reactive pulsed DC magnetron sputtering process. In this study, DLC coatings can be compared with each other as a tool to understand the role of carbon in Ti/WC films.

### 3.3. Residual Stress in Ti/WC Thin Films

**Figure 6:**
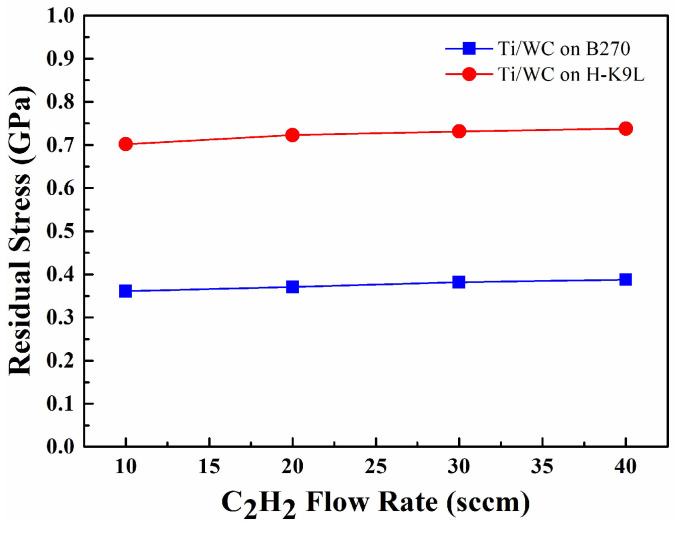
Residual stress of Ti/WC thin films deposited on B270 and H-K9L substrates.

The most applied technique to evaluate the residual stress in a thin film deposited on a substrate is the so-called laser beam deflection technique. Due to its simplicity and time-consuming procedures, in this study, the radius of curvature is measured using an optical interferometer combined with the fast Fourier transform method, and then converted into residual stress according to the Stoney equation [27]. Two kinds of glass substrates (B270 and H-K9L glasses, 25 mm in diameter) were used for stress measurement. The residual stress of the film is evaluated by the curvature of the substrate before and after deposition. In the stress measurement, the residual tensile stress in Ti metal film (thickness 0.69 μm) was 0.576 GPa for the B270 substrate and 0.570 GPa for the H-K9L substrate. Stress measurements of Ti/WC films deposited on B270 substrates revealed that the tensile stress of the Ti/WC films increased continuously with the increase in the C_2_H_2_ flow rate, and the stress value changed from 0.361 GPa to 0.405 GPa, as shown by the blue line in Figure 6. On the other hand, stress measurements of Ti/WC films deposited on H-K9L substrates revealed that the tensile stress of the Ti/WC films increased continuously with the increase in the C_2_H_2_ flow rate, and the tensile stress value changed from 0.702 GPa to 0.752 GPa, as shown by the red line in Figure 6. As the C_2_H_2_ flow rate increases, more carbon is incorporated into the Ti/WC thin films. The incorporation of carbon can cause changes in the film’s microstructure. For instance, a higher C_2_H_2_ flow rate could lead to the formation of more fine-grained or amorphous structures in the films, which might cause internal stresses as the thin film tries to accommodate these microstructural changes. The more compact structure at higher flow rates can generate more tensile stress due to the contraction of the film during deposition. Furthermore, for Ti/WC thin films, the tensile stress can stretch the atomic bonds, particularly in the Ti-C or WC phases. The elongation of this bond causes a reduction in the lattice vibration frequency, resulting in a shift in the Raman peaks toward lower wavenumbers. This shift is a direct consequence of the tensile strain in the film. The shift of the strong Raman peaks to lower wavenumbers in the Ti/WC films indeed indicates tensile stress and is consistent with the results shown in Figure 5.

This tensile stress behavior indicates that the first Ti deposited layer causes a significant deformation of the substrate, while the subsequent deposited WC layer results in a more relaxed thin film. Bilek et al. [31,32] reported that energy particles cause stress relaxation. The high-energy particles allow for some degree of atomic relaxation, thus reducing film stress. The results show that there is a clear correlation between the mechanical residual stress state and the Ti/WC films deposited on different substrates. The H-K9L substrate has a larger Young’s modulus (i.e., 79 GPa) and strain than the B270 substrate, which results in larger residual stress in the Ti/WC films. This study highlights the importance of controlling residual stress during deposition on different glass substrates to optimize thin film properties for specific applications. Baba et al. [33] reported a two-layer approach to reduce residual stress by adding a lower-hardness intermediate layer. The stress of the resulting bilayer film can be reduced to below 1.4 GPa while maintaining the high hardness of the top layer. After comparison, it is found that the Ti/WC double-layer coating structure proposed in this study can achieve even lower stress.

**Figure 7:**
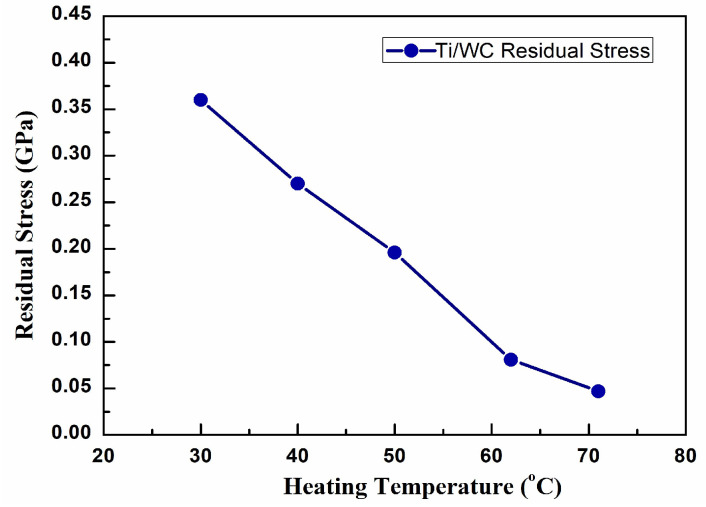
Residual stress vs. heating temperature for Ti/WC thin films.

Thermal stress poses a significant problem for DLC and diamond films due to the low thermal expansion coefficient of diamond (above 1.0 × 10^−6^ °C^−1^). However, when diamond films are deposited on substrates at high temperatures, the subsequent cooling to room temperature enhances the bonding between the film and the substrate as compared to diamond over-layers [34]. The diamond film bonds to the substrate and the interface area changes to accommodate the orientation of the substrate, resulting in compressive stress on the film. This study presents a thermal stress evaluation method for Ti/WC thin films. Figure 7 shows the residual stress of Ti/WC deposited on B270 glass substrate as a function of heating temperature in the range of 30–70 °C. The stress value is changed from 0.36 GPa to 0.047 GPa during the heating process. The results of residual stress versus heating temperature show that Ti/WC thin films deposited on B270 glass substrates have lower tensile stress after film deposition. One of the major obstacles that can lead to functional failure of Ti/WC coatings is residual stress, which can cause significant deformation and even lamination, buckling, and cracking. Thermal stress can be reduced by choosing a smaller difference in thermal expansion coefficients between the substrate and the thin film. The optical interferometry analysis revealed significant variations in residual stress levels among the deposited films. Thin films deposited at higher temperatures exhibited compressive stresses, while those deposited at lower temperatures showed tensile stresses.

### 3.4. Surface Roughness of Ti/WC Thin Films

**Figure 8:**
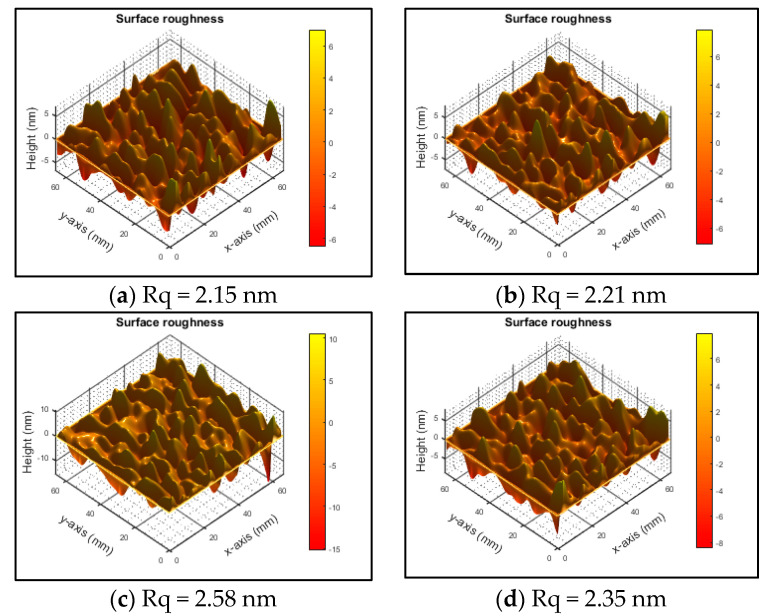
3D surface morphology and corresponding RMS roughness of Ti/WC thin films: (**a**) A6C1; (**b**) A6C2; (**c**) A6c3; (**d**) A6C4.

**Figure 9:**
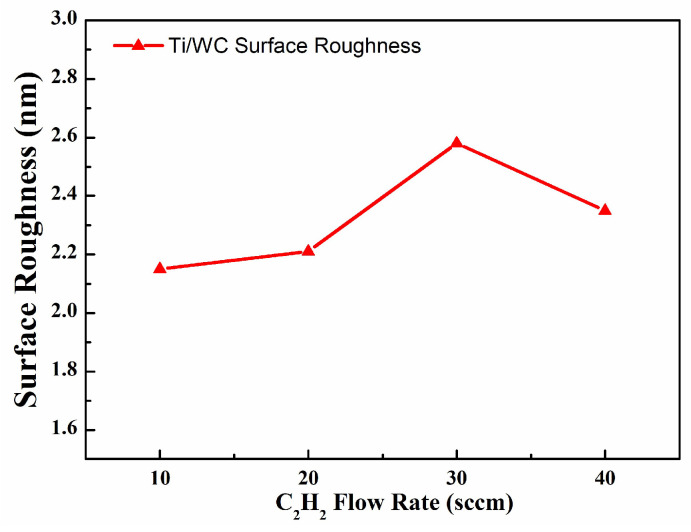
Surface roughness of Ti/WC thin films varied with C_2_H_2_ flow rate.

The root mean square (RMS) surface roughness of the Ti/WC thin films was measured using a homemade Linnik microscopic interference system and subjected to optical interference image processing analysis. Figure 8 shows the surface morphology and roughness measurement results of Ti/WC films deposited on B270 glass with different carbon contents, where the RMS surface roughness values are 2.15 nm, 2.21 nm, 2.57 nm, and 2.38 nm, respectively. It is evident that the surface roughness of Ti/WC thin films increases first and then decreases slightly as the carbon concentration rises. The increase in carbon content may result in a rougher film surface. Higher carbon contents may lead to uneven distribution of dopant atoms or cause internal stress in the film structure, leading to surface irregularities, grain boundaries, or even phase separation, thereby increasing the surface roughness. The surface roughness of Ti/WC films increases with the C_2_H_2_ flow rate from 10 sccm to 30 sccm, then slightly decreases from 30 sccm to 40 sccm, as shown in Figure 9.

### 3.5. X-Ray Diffraction (XRD) Pattern Analysis of Ti/WC Thin Films

**Figure 10:**
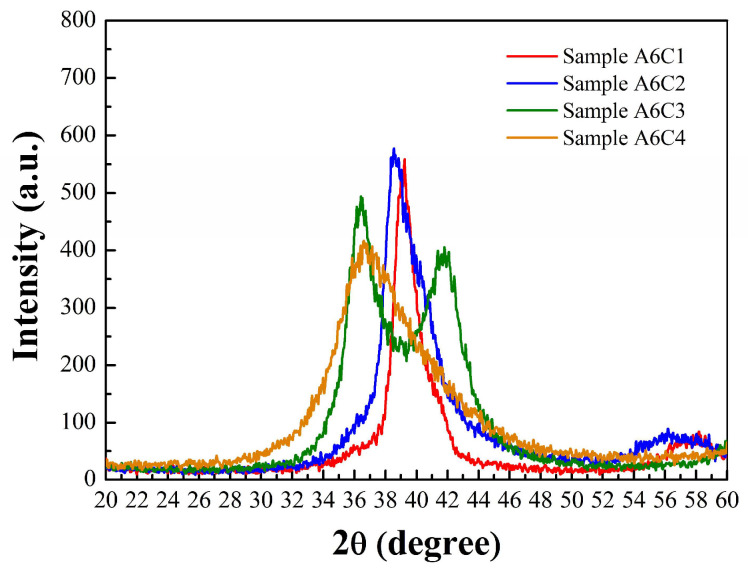
X-ray diffraction (XRD) pattern of Ti/WC thin films.

Glancing Incidence X-Ray Diffraction (GIXRD) was performed using a high-resolution X-ray diffractometer (Bruker, D8 Discover, Billerica, MA, USA). The GIXRD patterns were recorded at a glancing angle of 1° using Cu-Kα radiation having a wavelength equal to 1.54 Å, at 45 kV generator voltage and 40 mA tube current, over a 2θ range of 20° to 60°. Figure 10 shows the XRD peaks of four WC thin films observed at 2θ values of 39.08° (sample A6C1), 38.56° (sample A6C2), 36.5° (sample A6C3), and 36.74° (sample A6C4), respectively. It can be observed that the Ti/WC peak’s intensity and broadness decrease with the increase in C_2_H_2_ flow rates. According to Bragg’s law, when X-rays with a fixed wavelength are directed at a sample and the angle θ between the incident X-ray beam and a crystal plane satisfies the condition 2dsinθ = mλ, where d is the lattice spacing between crystal planes, λ is the wavelength, and m is an integer, diffraction occurs. The XRD pattern shows that the lattice spacing (d) increases with the decrease in 2θ values of Ti/WC thin films. A tensile stress (lattice stretching) increases the lattice spacing (d), thus shifting the diffraction peak to a lower 2θ value (decreasing angle). In other words, as the C_2_H_2_ flow rate increases, the tensile stress of the Ti/WC film also increases. The results of qualitative analysis of residual stress using XRD diffraction peaks are consistent with the results of residual stress of Ti/WC films measured using the optical interferometer.

## 4. Conclusions

This work explores the effects of different C_2_H_2_ flow rates on the optical properties, surface roughness, and residual stress of Ti/WC thin films deposited by the reactive pulsed DC magnetron sputtering technique. From the measurement results of Ti/WC thin films, we known that with the increase in the C_2_H_2_ flow rate, the average reflectance in the visible light spectrum (400–800 nm) decreases, and the infrared reflectance (2.5–25 μm) also decreases with the increase in the C_2_H_2_ flow rate. The surface roughness of the films initially increases with increasing C_2_H_2_ flow rates, but after reaching a peak, it decreases slightly. The tensile stress of the Ti/WC films increases with the C_2_H_2_ flow rate. Additionally, the Ti/WC films exhibit lower tensile stress after deposition when subjected to higher heating temperatures. The study highlights how adjusting the C_2_H_2_ flow rate can optimize the optical performance, surface characteristics, and stress behavior of Ti/WC thin films. In addition, the measurement results show that the Ti/WC film deposited on different glass substrates also has a significant impact on the performance of the thin film. In particular, the characteristic peaks associated with the WC phase (which are often observed around 1340 cm^−1^ and 1600 cm^−1^) can shift due to the internal stress within the thin film material. For the Raman spectra measurement of Ti/WC films, a microcrystalline diamond peak at 1100 cm^−1^ is found. The shift of strong Raman peaks to lower wavenumbers in Ti/WC films is indeed related to tensile stress. The findings are critical for tailoring thin film properties for specific applications, such as optical hard coatings and protective layers, by adjusting the deposition conditions to meet performance criteria. This study concluded that the C_2_H_2_ flow rate plays a crucial role in tuning the properties of Ti/WC thin films and that optimizing this parameter is important for enhancing their performance in different applications.

In summary, this paper is devoted to a comprehensive study of the effects of C_2_H_2_ flow rate on various properties of Ti/WC films. It provides a comprehensive understanding of how C_2_H_2_ flow rate affects several important properties, thus helping to optimize Ti/WC films for a wide range of applications. This provides valuable insights into the design of thin films with customized properties, which have important implications for applications that rely on coatings with specific optical, mechanical, and surface properties.

## Figures and Tables

**Table 1:** Sputtering conditions for the deposition of Ti/WC thin films.

Thin Film Samples	Ar Flow Rate (sccm)	C_2_H_2_ Flow Rate (sccm)	Processing Time (min)	Initial Pressure (Pa)	Working Pressure (Pa)	Substrate Temperature (°C)
A6C1	60	10	Ti: 40; MF: 40	1.5 × 10^−5^	0.25	80 ± 5
A6C2	60	20	Ti: 40; MF: 40	1.5 × 10^−5^	0.25	80 ± 5
A6C3	60	30	Ti: 40; MF: 40	1.5 × 10^−5^	0.25	80 ± 5
A6C4	60	40	Ti: 40; MF: 40	1.5 × 10^−5^	0.25	80 ± 5

**Table 2:** Physical parameters of different glass substrates.

Glass Substrates	B270	H-K9L
Thermal expansion coefficient (°C^−1^)	8.2 × 10^−6^	7.6 × 10^−6^
Young’s modulus (GPa)	71.5	79
Poisson’s ratio	0.219	0.214
Thickness (mm)	1.5	1.5

## Data Availability

The original contributions presented in this study are included in the article. Further inquiries can be directed to the corresponding author.
